# Effects of trimetazidine on periprocedural microRNA-21 expression by CD4+ T lymphocytes in patients with unstable angina pectoris

**DOI:** 10.18632/oncotarget.20975

**Published:** 2017-09-18

**Authors:** Qiang Su, Lang Li, Jinmin Zhao, Yuhan Sun, Huafeng Yang

**Affiliations:** ^1^ Department of Cardiology, The First Affiliated Hospital of Guangxi Medical University, Nanning 530021, China; ^2^ Department of Trauma Orthopedic and Hand Surgery, The First Affiliated Hospital of Guangxi Medical University, Nanning 530021, China; ^3^ Guangxi Key Laboratory of Regenerative Medicine, Guangxi Medical University, Guangxi 530021, China

**Keywords:** unstable angina pectoris, trimetazidine, CD4+ T lymphocyte, microRNA-21, PDCD4

## Abstract

**Objective:**

Post-percutaneous coronary intervention (PCI) myocardial injury is related to the CD4+ T lymphocyte-mediated inflammatory response. microRNA-21 expression is associated with CD4+ T lymphocyte activation. The pre-PCI use of trimetazidine prevents periprocedural myocardial injury and reduces inflammatory cytokine levels. This study aimed to assess the effects of trimetazidine on periprocedural microRNA-21 expression by CD4+ T lymphocytes in patients with unstable angina pectoris.

**Methods:**

A total of 252 patients with unstable angina pectoris were randomized to the trimetazidine (60 mg/d, administered 3 days before PCI, n=128) and control (no trimetazidine, n=124) groups. Serum CK-MB, cTnI, and hs-CRP levels were tested pre-PCI and 16-24 h post-PCI. Peripheral blood CD4+ T lymphocytes were isolated by magnetic activated cell sorting. Quantitative polymerase chain reaction was used to assess microRNA-21 and PDCD4 mRNA expression levels in CD4+ T lymphocytes, and western blot was used to evaluate PDCD4 protein expression. Enzyme-linked immunosorbent assay was used to assess serum TNF-α and IL-10 levels.

**Results:**

Compared with the control group, the trimetazidine group had a lower frequency of patients with post-PCI serum CK-MB and cTnI levels higher than normal values; the trimetazidine group had also significantly lower serum hs-CRP and TNF-α levels, and higher IL-10 levels post-PCI. Finally, the trimetazidine group had significantly lower PDCD4 expression and higher microRNA-21 levels in CD4+ T lymphocytes post-PCI.

**Conclusions:**

Trimetazidine reduces the incidence of periprocedural myocardial injury, possibly by increasing microRNA-21 levels in CD4+ T lymphocytes and inhibiting PDCD4-mediated inflammatory response.

## INTRODUCTION

Periprocedural myocardial injury (PMI) is a frequent complication of percutaneous coronary intervention (PCI), especially among high-risk patients with acute coronary syndrome (ACS). Previous studies demonstrated that post-PCI activation of local CD4+ T lymphocytes and the subsequent myocardial inflammatory response probably play a role in PMI [1].

MicroRNAs (miRNAs) are endogenous non-coding small RNA molecules of 18-25 nucleotides. They are important post-transcriptional regulatory genes widely found in the non-coding regions of the genome, regulating cell proliferation, cell differentiation, inflammation, and apoptosis [2, 3]. Lu *et al*. [4] reported that miRNA-21 downregulation in CD4+ T lymphocytes enhanced Th1 cell-mediated inflammatory response and reduced Th2 cell-secreted anti-inflammatory cytokines, indicating that miRNA-21 upregulation may inhibit the inflammatory response mediated by over-activated Th1 cells. miRNA-21 inhibits its target gene (programmed cell death 4 (PDCD4) protein) and the corresponding signaling pathway, controlling the differentiation, activation, and functionalization of Th1 cells [4]. In addition, miRNA-21 upregulation is likely to reduce the secretion of the proinflammatory cytokine tumor necrosis factor (TNF)-α and promote that of the anti-inflammatory cytokine interleukin (IL)-10, suppressing Th1 cell-mediated inflammatory response [5, 6]. We hypothesized that post-PCI myocardial injury may be alleviated by miRNA-21 upregulation in CD4+ T lymphocytes, improving patient prognosis.

Trimetazidine (TMZ), or 1-(2,3,4-trimethoxybenzyl) piperazine dihydrochloride, is a piprazine derivative that selectively inhibits fatty acid oxidation and stimulates glucose oxidation. TMZ improves myocardial blood supply by directly altering cardiac metabolic substrates and optimizing myocardial metabolism [7]. Labrou *et al*. [8] reported that the pre-PCI oral administration of TMZ significantly decreased post-PCI cTnI levels, alleviated PMI, and improved left ventricular function. Our preliminary studies demonstrated that pre-PCI TMZ is effective in lowering serum hs-CRP levels and reducing PMI, although the underlying mechanisms remain unclear.

Recently, TMZ was found to upregulate miRNA-21 in cardiomyocytes and mitigate myocardial ischemia/reperfusion injury [9]. We inferred that the same mechanisms may play a role in the protective effects of TMZ in PMI. In this study, patients with unstable angina pectoris (UAP) were administered TMZ before PCI. Post-PCI expression levels of miRNA-21 and the target gene PDCD4 were assessed in CD4+ T lymphocytes. Serum levels of cytokines (including IL-10, TNF-α, and high-sensitivity C-reactive protein (hs-CRP)) and cardiac markers of myocardial necrosis (including creatine kinase-MB (CK-MB) and cardiac troponin I (cTnI)) were also evaluated. This study may help clarify the cardioprotective mechanisms of TMZ during the periprocedural period, and provide a new approach for preventing and treating coronary artery diseases as well as PMI.

## RESULTS

### Baseline characteristics of the patients

The TMZ group was not significantly different from the control group in terms of age, sex, smoking status, diabetes mellitus, dyslipidemia, hypertension, history of cardiovascular disease, and medication use (all *P*>0.05). The two groups were comparable in terms of coronary lesion location, stenosis severity, stenosis number, and stent number (all *P*>0.05) (Tables 1 and 2).

**Table 1:** Clinical characteristics of patients

Parameters	Trimetazidine (n = 128)	Control (n = 124)	P
Male	100 (78)	93 (75)	0.331
Age, years	65.18±10.97	63.87±10.13	0.326
BMI, kg/m^2^	23.8 ±2.7	24.1 ±3.2	0.565
Hypertension	59 (46)	52 (42)	0.295
Diabetes mellitus	29 (23)	26 (21)	0.432
Dyslipidemia	45 (35)	37 (30)	0.222
Previous AP	27 (21)	21 (17)	0.248
Current smoker	52 (41)	46 (37)	0.328
Systolic BP, mmHg	138.02±17.11	135.24±15.01	0.192
Diastolic BP, mmHg	82.98±11.67	78.19±10.88	0.483
LVEF, %	52.26±10.74	53.41±11.33	0.563
*Treatment received*			
Aspirin	128 (100)	124 (100)	NS
Clopidogrel sulfate	128 (100)	124 (100)	NS
Statins	128 (100)	124 (100)	NS
ACEIs/ARBs	105 (82)	93 (75)	0.114
Beta-blockers	74 (58)	67 (54)	0.317
Calcium antagonists	31 (24)	26 (21)	0.321
Nitrates	55 (43)	46 (37)	0.205
GPIIb/IIIa inhibitors	7 (5.5)	6 (4.8)	0.106

Values are numbers of patients with percentages in parentheses, or mean ± SD. BMI = Body mass index; AP = angina pectoris; BP = blood pressure; LVEF = left ventricular ejection fraction; ACEIs = angiotensin-converting enzyme inhibitors; ARBs = angiotensin-receptor blockers; GPIIb/IIIa = glycoprotein IIb/IIIa; NS = Not significant.

**Table 2:** Treatment characteristics of patients

Parameters	Trimetazidine (n = 128)	Control (n = 124)	P
Vessel treated			
Left anterior descending	97 (76)	98 (79)	0.647
Left main	14 (11)	16 (13)	0.312
Left circumflex	54 (42)	47 (38)	0.341
Right coronary artery	70 (55)	61 (49)	0.127
One-vessel Intervention	73 (57)	73 (59)	0.402
Two-vessel Intervention	42 (33)	35 (28)	0.262
Multi-vessel Intervention	13 (10)	16 (13)	0.387
Stents per patient	1.72 ± 0.84	1.56 ± 0.79	0.622
Total stent length	25.17 ± 7.91	24.08 ± 8.13	0.193
Stent diameter	3.00 ± 0.44	3.00 ± 0.51	0.927
Stent deployment pressure	13.16 ± 2.68	12.63 ± 2.47	0.152
Drug-eluting stent	128 (100)	124(100)	NS
Dissection	0 (0)	0 (0)	NS
TIMI flow 3 before PCI	113 (88.3)	113 (91.1)	0.551
TIMI flow 3 after PCI	124 (96.9)	117 (94.4)	0.738

Values are number of patients with percentage in parentheses, or mean ± SD; TIMI = thrombolysis in myocardial infarction; NS = Not significant.

### Serum cardiac markers and hs-CRP

Baseline serum CK-MB and cTnI levels were normal and not significantly different between the TMZ and control groups. Compared with the control group, the TMZ group had a significantly lower proportion of patients with post-PCI cTnI (38.2% vs. 70.4%, *P*<0.05) and CK-MB (18.7% vs. 44.2%, *P*<0.05) levels higher than the reference values at 16-24 h.

Baseline serum hs-CRP levels were not significantly different between the TMZ and control groups (1.99±0.82 mg/l vs. 1.63±0.67 mg/l, *P*>0.05). Both groups had significantly elevated hs-CRP levels post-PCI compared with baseline values, with significantly higher levels found in the control group (5.85±1.32 mg/l *vs*. 9.63±0.67 mg/l, *P*<0.05).

### Serum TNF-α and IL-10 levels

Baseline serum TNF-α levels were not significantly different between the TMZ and control groups (7.14±0.77 pg/ml vs. 7.51±0.82 pg/ml, *P*>0.05). TNF-α levels were significantly increased in both groups at 16-24 h post-PCI, with higher amounts detected in the control group (13.18±2.62 pg/ml vs. 19.84±2.91 pg/ml, *P*<0.05).

Baseline serum IL-10 levels were not significantly different between the TMZ and control groups (6.27±0.93 pg/ml *vs*. 6.41±0.86 pg/ml, *P*>0.05). IL-10 levels were significantly increased at 16-24 h post-PCI, with higher values detected in the TMZ group (15.13±2.36 pg/ml vs. 11.87±1.95 pg/ml, *P*<0.05).

### Changes in miRNA-21 levels and PDCD4 mRNA expression in CD4+ T lymphocytes

**Figure 1:**
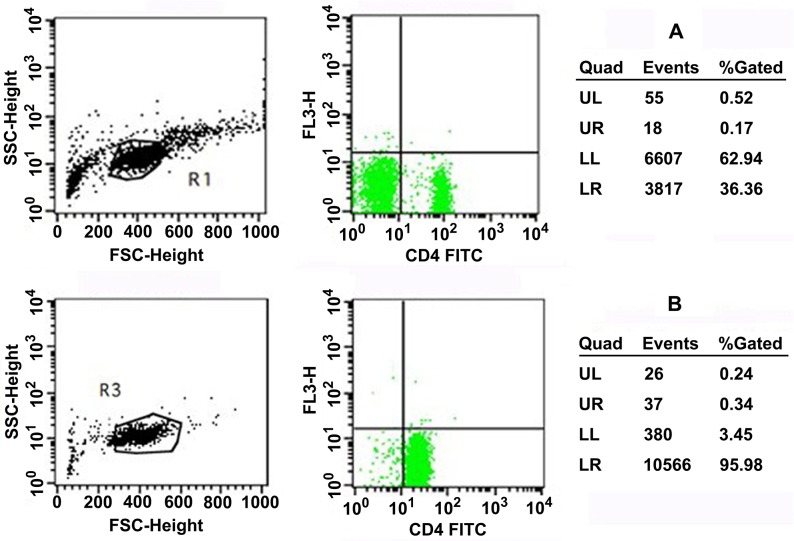
**(A)** Isolation of CD4+ T cells isolated from mononuclear cells. Pure CD4+ T cells are gated in LR. **(B)** Purity and viability of CD4+ T cells. Pure CD4+ T cells are gated in LR. UL = Upper left; UR = upper right; LL = lower left; LR = lower right.

**Figure 2:**
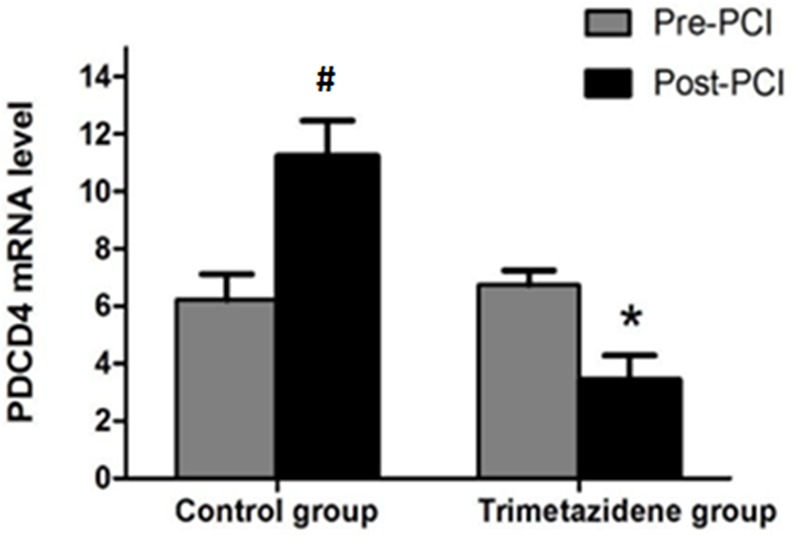
Fluorescence-based RT-PCR was used to detect PDCD4 gene expression ^#^*P*<0.05 vs. the control group (n=124, pre-PCI) as assessed by the paired t test. ^*^P<0.05 vs. the trimetazidine group (n=128, pre-PCI) as assessed by the paired t test.

**Figure 3:**
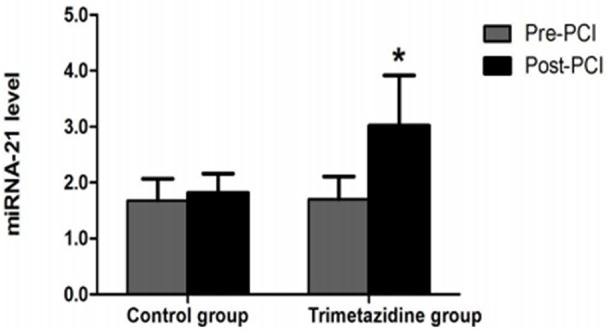
Fluorescence-based RT-PCR was used to assess miRNA-21 levels ^*^P<0.05 vs. the trimetazidine group (n=128, pre-PCI) as assessed by paired t test.

CD4+ T lymphocytes were sorted using magnetic beads and had >90% purity (Figure 1). Baseline miRNA-21 levels and PDCD4 mRNA expression were not significantly different between the two groups (*P*>0.05). The TMZ group had significantly reduced PDCD4 mRNA expression and increased miRNA-21 expression post-PCI (*P*<0.05). The control group had significantly higher PDCD4 mRNA levels post-PCI (*P*<0.05) (Figure 2), but miRNA-21 expression was not significantly changed (*P*>0.05) (Figure 3).

### Changes in PDCD4 protein levels in CD4+ T lymphocytes

**Figure 4:**
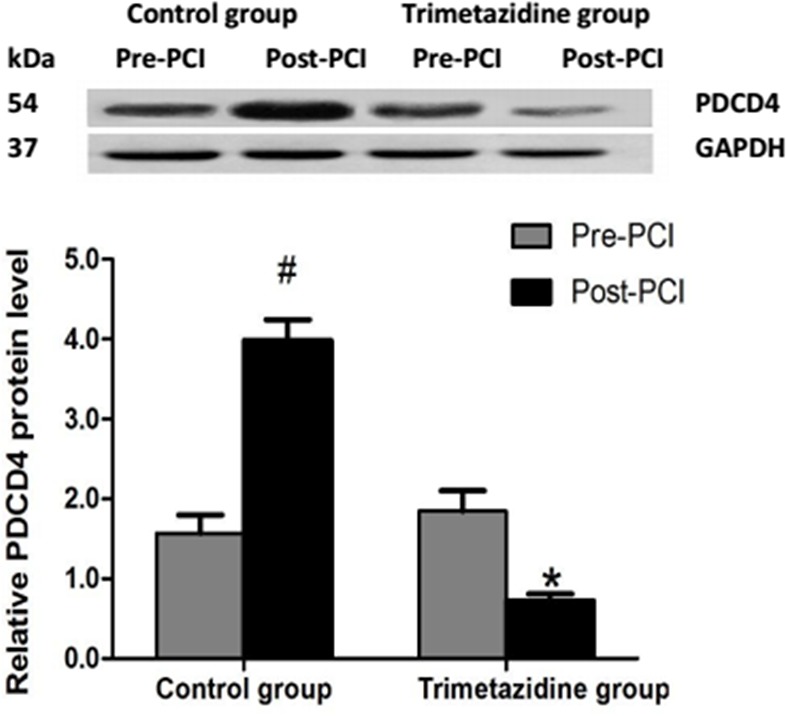
Western blot analysis of PDCD4 protein expression Relative expression levels of PDCD4 were determined by western blot. ^#^*P*<0.05 vs. the control group (n=124, pre-PCI); ^*^P<0.05 vs. the trimetazidine group (n=128, pre-PCI) as assessed by the paired t test.

Baseline relative PDCD4 protein levels were not significantly different between the two groups (*P*>0.05). The TMZ group had significantly lower relative PDCD4 protein amounts post-PCI (*P*<0.05). The control group had significantly higher relative PDCD4 protein levels post-PCI (*P*<0.05) (Figure 4).

### Correlations of CD4+ T lymphocyte PDCD4 levels with inflammation markers and miRMA-21

PDCD4 levels were positively correlated with hs-CRP (r=0.714, *P*<0.01) and TNF-α (r=0.634, *P*<0.01) levels, and negatively correlated with miRNA-21 (r=-0.679, *P*<0.01) and IL-10 (r=-0.525, *P*<0.01) levels.

## DISCUSSION

PMI is a common complication of elective PCI, occurring in 30-70% of cases, as indicated by elevated CK-MB or cTnI levels [10]. Previous studies demonstrated that PMI significantly increases long- and short-term cardiovascular adverse events and leads to unfavorable prognosis. Meanwhile, the severity of PCI-induced myocardial injury is highly correlated with the incidence of long-term cardiovascular adverse events [11, 12]. As shown above, serum CK-MB and cTnI levels were elevated post-PCI, accompanied by changes of hs-CRP, TNF-α, and IL-10 amounts, indicating that the inflammatory response likely plays a role in PMI. Pre-PCI oral administration of TMZ may significantly improve PMI by reducing serum hs-CRP and TNF-α levels and elevating IL-10 amounts. TNF-α is an important pro-inflammatory cytokine that suppresses cardiac contractility and activates neutrophils. It stimulates neutrophils and endothelial cells to excessively express adhesion molecules, leading to tissue neutrophil accumulation and subsequent tissue impairment [13]. IL-10 is an anti-inflammatory cytokine secreted by Th2 cells. Derkacz *et al*. [14] reported that elevated serum IL-10 levels is associated with reduced incidence of PMI. Our results demonstrated that TMZ may alleviate PMI through its anti-inflammatory effects.

PMI results from multiple factors such as microvascular embolization, coronary artery spasm, microcirculation hypoperfusion, and ischemia-reperfusion injury. Activation of cardiac CD4+ T lymphocytes and the subsequent myocardial inflammation play crucial roles in PMI. We previously reported that the PDCD4 protein is involved in post-PCI CD4+ T lymphocyte-mediated inflammatory response and subsequent PMI by increasing serum TNF-α levels and reducing IL-10 levels [15]. Recent studies have shown that miRNAs regulate the development of the immune system and immune response, participating in immunity-mediated diseases. miRNAs exert their functions mainly through the post-transcriptional regulation of protein synthesis [16–18]. Since PDCD4 is one of the key target genes of miRNA-21, it is possible to regulate PDCD4 protein synthesis by controlling miRNA-21 expression. Garchow *et al*. [19] showed that miRNA-21 downregulation increases PDCD4 expression levels in CD4+ T lymphocytes, indicating that miRNA-21 negatively regulates PDCD4 expression and reduces the inflammatory response. Therefore, we inferred that a drug likely to affect miRNA-21 expression would also likely decrease CD4+ T lymphocyte-mediated inflammatory response post-PCI and result in reduced PMI incidence.

Recently, the cardioprotective effects of TMZ have been attributed to miRNA-21 regulation. Ma *et al*. [9] reported that TMZ improves myocardial ischemia-reperfusion injury by upregulating miRNA-21 and modulating the Akt and Bcl-2/Bax pathways. Yang *et al*. [20] showed that TMZ exerts its cardioprotective effects by suppressing hypoxia-reperfusion cardiomyocyte apoptosis and upregulating miRNA-21. As shown above, pre-PCI administration of TMZ significantly upregulated miRNA-21, effectively reduced PDCD4 secretion by CD4+ T lymphocytes, lowered serum TNF-α levels, and increased IL-10 levels, resulting in decreased PMI incidence. Interestingly, miRNA-21 correlated with PDCD4, which was also correlated with TNF-α and IL-10, as shown above. These findings confirmed the notion that TMZ exerts cardioprotective effects by regulating periprocedural miRNA-21 expression to reduce PMI.

Limitations: Clinically important endpoints, such as death, myocardial infarction, and revascularization rate, were not recorded. In future studies, more clinically relevant endpoints will be considered. PDCD4 may be a downstream target of other factors (e.g., TGF-β, IL-2, IL-15, IL-12, etc.) [21, 22], but the present study did not examine all these factors that may be associated with periprocedural myocardial injury. On the other hand, miRNA-21 levels were only increased in the TMZ group and this suggests that the increase in miRNA-21 could be induced by TMZ. Additional studies are necessary to examine these factors. Furthermore, no mechanistic studies could be made in the present preliminary study. Finally, large-scale randomized controlled trials are warranted to validate these findings.

In conclusion, administration of 60 mg/d TMZ for 3 days pre-PCI effectively reduces hs-CRP levels and PMI incidence by upregulating miRNA-21 in CD4+ T lymphocytes and suppressing PDCD4-mediated inflammatory response. This mechanism may help discover a novel approach for PMI prevention and treatment.

## MATERIALS AND METHODS

### Study design and patients

From February 2011 to July 2015, 252 patients with UAP (193 males and 59 females) were randomized to the TMZ (20 mg tid TMZ, for 3 days before PCI, n=128) and control (no TMZ, n=124) groups, according to a random number table. All patients with UAP had coronary stenosis confirmed by coronary angiography, and had indications for PCI. Cardiac makers (CK-MB and cTnI) were negative in these patients at baseline. No patients had contraindications to statins. All patients had either new-onset angina, worsening effort angina, or resting angina. The exclusion criteria were: 1) severe infections or tumors; 2) severe liver or renal dysfunction; 3) allergy to TMZ; 4) stroke; 5) left ventricular ejection fraction <30%; or 6) emergency PCI. The study protocol complied with the human trial regulations developed by the Institutional Review Board of Guangxi Medical University. Written informed consent was obtained from all participants.

### Sample collection

Peripheral venous blood (20 ml) was drawn before PCI and at 16-24 h after PCI (mean of 20 h in both groups, P>0.05). One ml of blood was kept at room temperature for 20 min and centrifuged at 2000 rpm for 10 min; the resulting serum was assessed for CK-MB, cTnI, and hs-CRP levels. Serum TNF-α and IL-10 levels were measured with specific ELISA detection kits (eBioscience, USA), according to the manufacturer's instructions. The remaining 19 ml of peripheral venous blood were added with heparin for cell isolation.

### Cell isolation

Peripheral blood mononuclear cells (PBMCs) were isolated by Ficoll-Paque density gradient centrifugation. PBMCs were then resuspended in 1 ml of RPMI 1640 culture medium (Hyclone, USA), and 10 μl of PBMC suspension were added to 90 μl of phosphate-buffered saline for cell counting. The remaining PBMCs were sorted for CD4+ T lymphocytes, according to the instructions of the Dynabeads FlowComp Human CD4 kit (Dynal, Norway). Briefly, the beads were first washed according to the manufacturer's protocol. Then, 500 μL of cells were added with 25 μL of the FlowComp Human CD4 antibody, mixed well, and incubated 10 min at 4°C. The cells were washed with 2 mL of isolation buffer and centrifuged at 350 g. The supernatant was discarded and 75 μL of FlowComp Dynabeads were added. The cells were incubated at room temperature for 15 min under gentle agitation. One mL of isolation buffer was added, mixed for 2-3 s, and the tube was placed in a magnet for 2 min. The supernatant was discarded with the tube still in the magnet. Washing was repeated twice. The cells were released using the release buffer and the beads were caught using the magnet. The supernatant containing the bead-free cells was transferred to a new tube. Stained cells were analyzed on a FACScan flow cytometer (BD Biosciences, USA) with a 488-nm argon laser and Cell Quest 3.1 software (BD Biosciences, USA). Lymphocytes were gated by forward scatter (FSC) and side scatter (SSC). The sorted lymphocytes were counted. Cell viability was evaluated by 0.4% Trypan blue staining. CD4+ T lymphocytes with cell viability >90% were used for the subsequent experiments.

### miRNA-21 levels and PDCD4 mRNA expression by quantitative fluorescence polymerase chain reaction (q-PCR) in CD4+ T lymphocytes

Total RNA was extracted from CD4+ T lymphocytes using TRIzol (KeyGen, China) according to the manufacturer's instructions, and quantitated using a Nanodrop system. RNA integrity was detected by 1% agarose gel electrophoresis. After adjusting the RNA concentration to 1 μg/μl, cDNA was synthesized using the Reverse Transcription Kit (Fermentas, Lithuania). PCR products were assessed by the SYBR Green I method, in a total reaction volume of 20 μl. Primers for miRNA-21 and the internal control U6 were provided by GeneCopeia (Maryland, USA; primers ID: hsmq-0057 and hsnRNAU6). The following primers (Sangon Biotech, China) were used: PDCD4, forward 5′-AAC TGT GCC AAC CAG TCC AA-3′ and reverse 5′-TCT TCT CAA ATG CCC TTT CAT C-3′; GAPDH forward 5′-GAG TCA ACG GAT TTG GTC GT-3′ and reverse 5′- GAC AAG CTT CCC GTT CTC AG-3′. The reaction system and parameters were based on the manufacturer's instructions. Each specimen was detected in multiple wells, and each reaction system included negative control wells. PCR products were sequenced. The final results were analyzed by the 2^−ΔΔCT^ method.

### Western blot for PDCD4 protein level assessment

Sorted CD4+ T lymphocytes were treated with 100 μl of protein lysis buffer and centrifuged at 12,000 r/min for 20 min at 4°C. Supernatants were transferred to clean centrifuge tubes and tested for protein concentration using the bicinchoninic acid assay, with GAPDH as internal control (Beyotime Institute of Biotechnology, China). Equal amounts of protein were separated by 12% sodium dodecyl sulphate polyacrylamide gel electrophoresis (SDS-PAGE) with 5% stacking gel, and transferred onto PVDF membranes by the semi-dry method for 40 min. The membranes were blocked in Tris-buffered saline-Tween containing 5% skimmed milk for 1 h, and incubated with the primary antibody (PDCD4, Abcam, USA; 1:2500) at 4°C overnight. After five washes of 5 min with TBST, the samples were incubated with 1:5000 infrared fluorescent secondary antibody (LI-COR, USA) at room temperature for 2 h. The membrane was then scanned on an Odyssey double-color infrared laser imaging system.

### Statistical analysis

Data were analyzed with SPSS 23.0 (IBM Corporation, Armonk, USA). Continuous data are presented as mean ± standard deviation. Inter-group comparison was performed by paired *t* test. Multiple group comparison was performed by one-way analysis of variance (ANOVA) with the Fisher's least significant difference post hoc test. Categorical data were expressed as frequency and constituent ratio, and compared by the chi-square test. *P*<0.05 was statistically significant.

## Abbreviations

ACS: acute coronary syndrome
ANOVA: analysis of variance
CK-MB: creatine kinase-MB
cTnI: cardiac troponin I
hs-CRP: high-sensitivity C-reactive protein
IL: interleukin
miRNA: microRNA
PMBCs: peripheral blood mononuclear cells
PCI: percutaneous intervention
PDCD4: programmed cell death 4
PMI: periprocedural myocardial injury
q-PCR: quantitative fluorescence polymerase chain reaction
SDS-PAGE: sodium dodecyl sulphate polyacrylamide gel electrophoresis
TMZ: trimetazidine
TNF-α: tumor necrosis factor α
UAP: unstable angina pectoris
